# The pattern of photosynthetic response and adaptation to changing light conditions in lichens is linked to their ecological range

**DOI:** 10.1007/s11120-023-01015-z

**Published:** 2023-03-28

**Authors:** Piotr Osyczka, Beata Myśliwa-Kurdziel

**Affiliations:** 1 Faculty of Biology, Institute of Botany, Jagiellonian University in Kraków, Gronostajowa 3, 30-387 Kraków, Poland; 2 Department of Plant Physiology and Biochemistry, Faculty of Biochemistry, Biophysics and Biotechnology, Jagiellonian University in Kraków, Gronostajowa 7, 30-387 Kraków, Poland

**Keywords:** Lichen ecophysiology, Tolerance range, Photobiont, Chlorophyll fluorescence, Fluorescence transient, Non-photochemical quenching

## Abstract

Epiphytic lichens constitute an important component of biodiversity in both deforested and forest ecosystems. Widespread occurrence is the domain of generalist lichens or those that prefer open areas. While, many stenoecious lichens find shelter only in a shaded interior of forests. Light is one of the factors known to be responsible for lichen distribution. Nevertheless, the effect of light intensity on photosynthesis of lichen photobionts remain largely unknown. We investigated photosynthesis in lichens with different ecological properties in relation to light as the only parameter modified during the experiments. The aim was to find links between this parameter and habitat requirements of a given lichen. We applied the methods based on a saturating light pulse and modulated light to perform comprehensive analyses of fast and slow chlorophyll fluorescence transient (OJIP and PSMT) combined with quenching analysis. We also examined the rate of CO_2_ assimilation. Common or generalist lichens, i.e. *Hypogymnia physodes*, *Flavoparmelia caperata* and *Parmelia sulcata*, are able to adapt to a wide range of light intensity. Moreover, the latter species, which prefers open areas, dissipates the excess energy most efficiently. Conversely, *Cetrelia cetrarioides* considered an old-growth forest indicator, demonstrates definitely lower range of energy dissipation than other species, although it assimilates CO_2_ efficiently both at low and high light. We conclude that functional plasticity of the thylakoid membranes of photobionts largely determines the dispersal abilities of lichens and light intensity is one of the most important factors determining the specificity of a species to a given habitat.

**Supplementary Information:**

The online version contains supplementary material available at 10.1007/s11120-023-01015-z.

## Introduction

Lichens constitute the symbiotic associations and result from interspecies relationships, the main components of which are a fungus and a phototroph, typically an alga and/or cyanobacterium (see Spribille et al. 2022). Combined into one functional organism, the components are involved in the growth of lichen thallus, and the vitality of one of the partners depends on the physiological condition of the other. In simple terms, fungal filaments organize the thallus structure and, by enclosing photobionts inside, providing them with shelter. In turn, the photosynthesis performed by a photobiont provides food for mycobiont since ribitol produced as a result of the photosynthesis process becomes a precursor for other sugar alcohols and fungal metabolites (Eisenreich et al. 2011; Lines et al. 1989). Green algae that belong to the class Trebouxiophyceae are the most common photobionts found in well over half of the lichen associations. They are rather indiscriminate in terms of their ability to establish intimate relations with different fungal partners (Blaha et al. 2006). Generally, lichenized *Trebouxia* is known, in a sense, to be a universal photosynthetic partner enclosed in thalli of lichens with different growth forms and various ecological preferences (Muggia et al. 2018).

The widespread distribution of lichens throughout the world and their ability to inhabit almost any kind of habitat is in part a reflection of their great diversity of symbiotic lifestyle combinations (Spribille 2018). Tree bark is one of the basic substrates for lichens in regions where the trees occur (Ellis 2012). Most epiphytic lichens demonstrate high specificity to a given habitat type, i.e. non-forested or forest area. There is also a relatively small group of non-specific lichens which are fairly indifferent to this respect (Kubiak and Osyczka 2020). Favorable and stable microclimatic conditions for lichen growth are ensured by forest communities, which frequently constitute refuge for sensitive species (Coppins and Coppins 2002). Strong confinement of some lichens to the forest interior is usually considered in the context of their high requirements for habitat humidity and low desiccation stress tolerance (Jonsson Čabrajić et al. 2010). As in the case of other factors, lichens that prefer or are sensitive to intense light conditions can be distinguished by inferring from their distribution pattern. However, the influence of light factor on performance of lichens in the environment is not fully elucidated (Gauslaa and Solhaug 1996; Gauslaa et al. 2012; Heber et al. 2000).

Light is the source of energy for the photosynthesis process. However, excess light leading to chlorophyll overexcitation is harmful to photosynthetic organisms (Müller et al. 2001). Protective mechanisms that are activated at different timescales include the dissipation of excess energy in the form of heat, redistribution of energy between photosystems and photoinhibition. These mechanisms, quite well understood in plants, algae and cyanobacteria, allow to adjust amount of energy supplied to the photosynthetic reaction centers, i.e. photosystem I (PSI) and photosystem II (PSII), to the efficiency of the photosynthetic electron transport chain (Magdaong and Blankenship 2018; Roach and Krieger-Liszkay 2019). Photoprotection mechanisms in lichens are much more complex, different in hydrated and dehydrated state and dependent on light intensity (Heber et al. 2000, 2006, 2010, 2011). Potential differences in this respect between lichens, which differ in terms of requirements for light conditions and inhabit sites with different insolation, have not yet been disclosed (see Beckett et al. 2021). 

The photosynthetic performance of photobionts guarantees vitality and growth of lichens in the habitat. Little is known about the response of the photosynthetic apparatus of lichen photobionts to changes or significant fluctuations in light intensity. The aim of our study was to determine the effect of light conditions on the characteristics of photosynthesis process in photobionts of lichens that demonstrate different ecological specificity. We intended to determine if there is a direct link between the response to light factor and habitat requirements of a given species. The research was based on healthy, well-hydrated lichen individuals collected directly from their natural habitat and the light parameter was the only factor modified during the examination. We hypothesized in general that the photosynthetic apparatus of photobionts in generalist lichens with a wide range of ecological tolerance demonstrate high functional plasticity in relation to changing light conditions; contrarily, stenoecious lichens that are closely associated with the shaded forest interior do not show such adaptive properties.

## Materials and methods

### Target species

Four epiphytic lichen species were selected for the study (abbreviations used later in the text are given in parentheses): *Cetrelia cetrarioides* (Duby) W.L. Culb. & C.F. Culb. (*Cet*; Fig. S1a), *Flavoparmelia caperata* (L.) Hale (*Fla*; Fig. S1b), *Hypogymnia physodes* (L.) Nyl. (*Hyp*; Fig. S1c), *Parmelia sulcata* Taylor (*Par*; Fig. S1d). The species differ in terms of light (insolation) requirements and are confined to old forest complexes to varying degrees; for general characteristics of the species, see Tables S1 (growth form, algal component, anatomy, ecology) and Table S2 (chlorophyll content).

### Sampling site

Lichen material were collected in the summer season of 2022 in the Bieszczady Mts. (Eastern Carpathians, SE Poland). The climate for this geographical region has been classified into Dfb type (the updated Köppen-Geiger classification, see Kottek et al. 2006). The mean annual values of basic climate parameters are as follows: temperature slightly above 10 °C; precipitation 900 mm (valleys) – 1200 mm (mountain ridges), relative humidity 77–84% (Nowosad 1995). Lichen specimens were obtained from one forest district area; therefore, it can be assumed that the populations of these species, except for light conditions (see Table S3), generally developed under the influence of the same external factors.

### Pre-treatment of lichen material

The analysis was performed on fresh lichen material, collected during a rainless period and transported to the lab in dry and dark conditions. The thalli were cleaned from macroscopic foreign materials adhering to their surface and sprayed until wet with rainwater harvested from the sampling area (pH 7.2, conductivity 27 μS cm^−1^). Then the thalli were placed into a ventilated chamber providing very high humidity (close to 100%) for 24 h to bring them to equilibrium state.

### Chlorophyll in vivo fluorescence and related parameters

Chlorophyll in vivo fluorescence emission is a non-invasive method to measure the photosynthetic activity of PSII (Murchie and Lawson 2013). Methods based on a saturating light pulse and on modulated light were applied. Comprehensive analyses of fast fluorescence kinetic (OJIP) and slow chlorophyll fluorescence transient (Kautsky curve) combined with quenching analysis were performed. Several key fluorescence parameters, namely F_O_, F_M_, F_V_/F_M_, PI_ABS_, ΔV_OJ_, ΔV_JI_, ΔV_IP_, V_K_/V_J_ (related to OJIP) and QY(max), QY, NPQ, qP and R_fd_ (related to modulated fluorescence), were analyzed in detail (see Table S4).

#### Fast fluorescence kinetics (OJIP)

An advanced continuous excitation chlorophyll fluorimeter Handy PEA + (Hansatech Instruments Ltd, Norfolk, England) was used. Ten fully hydrated samples of each species were inserted into leafclips with 4 mm diameter measuring aperture. Prior to measurements, the samples were adapted to darkness for 15 min, the reduced 4-min adaptation time was also practiced to test its applicability for further experiments. Chlorophyll fluorescence transients were induced by ultra-bright red-light (650 nm) provided by an array of three high-intensity LEDs. The light pulse intensity was 2400 µmol photons m^−2^ s^−1^ for 1 s, the gain of the PEA was 1.0.

#### Fluorescence imaging

Fluorescence imaging was performed using an Open FluorCam FC 800-O/1010 fluorimeter and a FluorCam7 software (PSI, Drásov, Czech Republic). Depending on the purpose and specifics of a given experiment, various standard protocols were applied with some modifications as described below.

Fully hydrated lichen thalli were divided into small separate fragments (1–1.5 cm^2^), placed evenly on round Petri dishes, sprayed again with water and covered with a lid to prevent water evaporation. They were pre-darkened for 15 min before measurements. Light curves were measured using a modified “Light Curve” protocols both for white and red actinic light. The time period between saturating pulses was prolonged to 4 min (Table S5). Saturating pulse intensity was 3000 µmol photons m^−2^ s^−1^. The intensity of the actinic light was changed after each saturating pulse within the following ranges: 8*–*1160 µmol photons m^−2^ s^−1^ for white light and 20*–*240 µmol photons m^−2^ s^−1^ for red light. All measurements were done for shutter set to “0” and sensitivity of 30*–*40%.

A modified “Quenching Protocol” was applied to reveal the process of lichen photobiont adaptation to a given light intensity. Measurements started from a saturating light pulse (3000 µmol photons m^−2^ s^−1^) followed by 1-min dark period. Then the samples were treated with white actinic light (8 or 500 µmol photons m^−2^ s^−1^) for 8 min followed by 100 s in the dark; the light intensities used relate to the light conditions for a forest interior and open area, respectively. The same intensity saturating pulses were applied at the end of the actinic light interval and at the end of final dark interval. Slow fluorescence transient (PSMT curve, see Papageorgiou et al. 2007) as well as the photochemical (qP) and non-photochemical quenching (NPQ) together with photosynthetic efficiency (QY(max) and QY) parameters and were analysed using a FluorCam7 software. The parameter qP was expressed here as 1-qP.

### Determination of CO_2_ assimilation

The average rate of CO_2_ exchange that corresponds to net photosynthesis was measured using the infrared gas analyser (maMoS200, Madur, Poland). The device was equipped with a transparent measuring chamber (50 mL) and operated in a closed system (for a detailed description of the method, see Field et al. 1989). The hydrated lobs of lichen thalli were evenly distributed inside the chamber over an area of approx. 36 cm^2^. The same laboratory conditions, including light intensities, were used as for the fluorescence quenching analysis. Three repetitions were performed for each species and light intensity. Prior to the measurements, the samples were treated with ambient light and the content of CO_2_ in the system was equilibrated to the range of 350*–*400 ppm. Changes in level of CO_2_ concentrations were recorded over a period of 25 min during alternating 200 s periods of light (8 or 500 µmol photons m^−2^ s^−1^, depending on the experiment) and darkness. Measurements performed under light conditions refer to net photosynthesis, i.e. the difference between the rate of CO_2_ assimilation in photosynthesis and the rate of CO_2_ release due to cellular respiration and photorespiration. During the darkness, the release of CO_2_ as a result of cellular respiration of both photobiont and mycobiont was measured. The slope coefficients of respective curves measured for light and dark periods were calculated to determine the rates of net photosynthesis and dark respiration, respectively. The rate values were expressed in ppm per minute. Finally, for each lichen species, the average gross CO_2_ assimilation in light (8 or 500 µmol photons m^−2^ s^−1^) was calculated as the difference between the rates of net photosynthesis and dark respiration,

### Fluorescence emission at 77 K

Low temperature fluorescence emission spectra were measured using Perkin-Elmer LS50B (Perkin Elmer, UK) fluorimeter equipped with the sample holder cooled with liquid nitrogen and dedicated for measurements at 77 K. Before proceeding to the measurement procedure, lichen thalli were incubated under conditions of dim scattered white light (less than 5 µmol photons m^−2^ s^−1^) and under high light (500 µmol photons m^−2^ s^−1^) for 30 min. Then, the thalli (5 replicates for each species and incubation) were homogenized in a sterile 1 mL of isotonic buffer (0.3 M sorbitol in 50 mM HEPES, pH 7.5; see Gasulla et al. 2010) using a ceramic mortar and pestle at the same light conditions as mentioned above. Aliquots of about 200 µL of homogenate were placed in sample tubes (7 cm × 0.3 cm). The samples were excited at 435 nm to record the emission spectra within the range of 640*–*790 nm. Excitation and emission slits were set to 5 nm and the speed was 300 nm/min. The tubes were slightly rotated and moved up/down between measurements and five spectra were obtained. Spectra were corrected for scattering and for wavelength dependence of photomultiplier. They were normalized to calculating the differential spectra.

### Data processing

The transient curves (OJIP) were plotted on a logarithmic time scale based on averaged data points (n = 10). The curves of variable fluorescence were calculated from chlorophyll fluorescence induction curves according to the formula: Vt = (F_t_ − F_O_) / (F_M_ − F_O_).

The statistical significance of differences was assessed using one-way ANOVA (p < 0.05) followed by Tukey’s HSD post-hoc test. A two-way analysis of variance (lichen species × light intensity) was also used. Before that, the normality distribution was checked using Kolmogorov–Smirnov test and Levene’s test was used to verify the homogeneity of variances; in necessary, the Box-Cox transformation was applied.

## Results

### Fast fluorescence kinetic: OJIP test

**Fig. 1:**
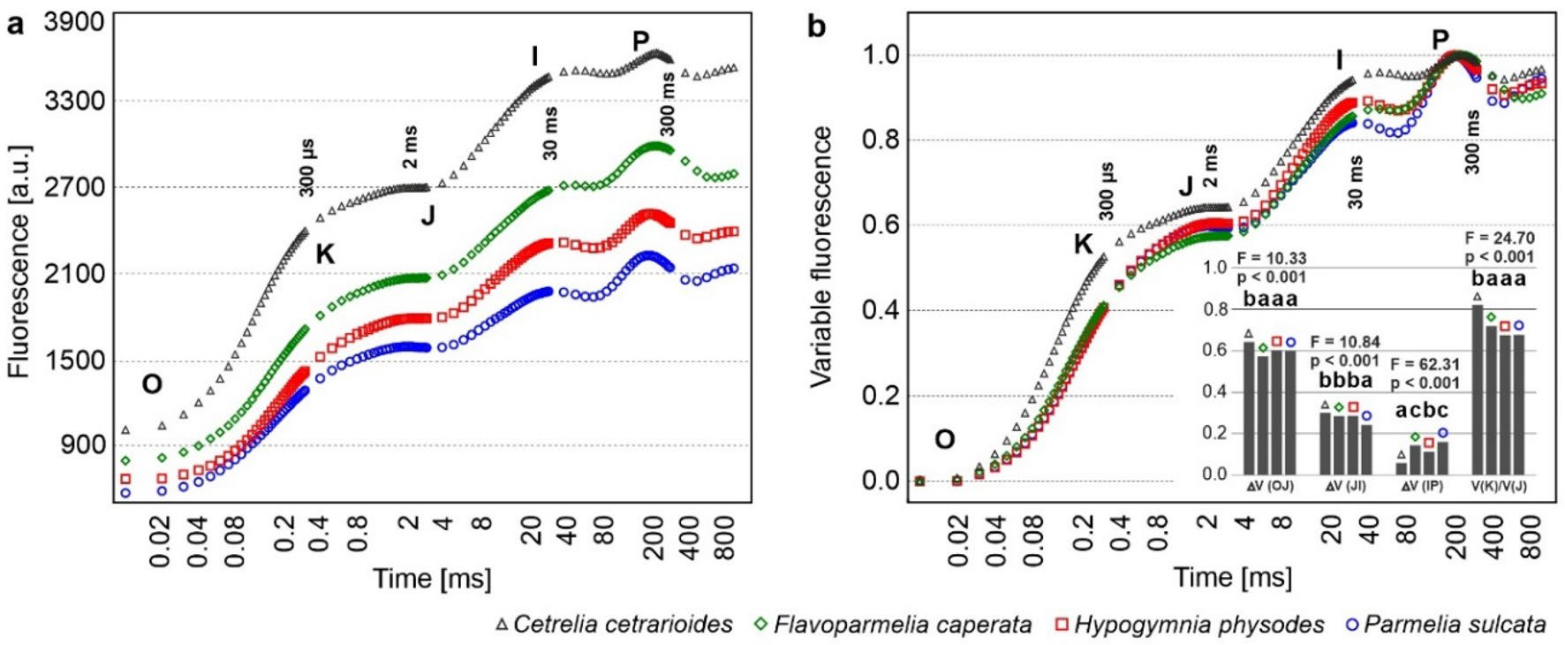
Chlorophyll *a* fluorescence induction curves (OJIP curves) for particular lichen species **(a)**. The curves of variable fluorescence calculated from chlorophyll *a* fluorescence induction curves **(b)**. The curves are plotted on a logarithmic time scale and based on the averaged data points (n = 10). The insertion shows the changes of amplitude of relative variable fluorescence in particular phases, the results of one-way ANOVA (F and p values) are provided, various letters indicate statistically significant differences (p < 0.05) according to the results of Tukey’s (HSD) post-hoc test

The OJIP test reveals the rapid response of a dark-adapted sample to a short and intense light pulse. The polyphasic increase of fluorescence intensity originates mainly from the PS II and reflects the reduction of the photosynthetic electron transport chain. Fast fluorescence transient curves obtained for all samples of each species revealed the characteristic sequence of OJIP steps (e.g. fluorescence rise from a minimum value O via the intermediate steps J and I to the maximum value P), which is typical of photosynthetic organisms, including healthy lichens (Fig. 1a). Nevertheless, they differ in terms of general shape and this applies especially to *Cet* where OJ and IP phases ran noticeable higher than in other species (Fig. 1b). Moreover, this species differed significantly from others in the changes of amplitude of relative variable fluorescence in OJ phase (ΔV_OJ_), IP phase (ΔV_IP_) and in terms of the calculated ratio V_K_/V_J_. The courses of the chlorophyll induction curves for *Fla* and *Hyp* were very similar (Fig. 1a and b). The curves for *Par* generally corresponded to the curves of these two lichens; however, the change of amplitude in JI phase noted for *Par* was the lowest among all species (Fig. 1b).

According to the measured fluorescence signals, the F_O_ and F_M_ values were always the highest for *Cet*, while the lowest for *Par* (Fig. 1a, Table S5). Although significant differences in the ratio F_V_/F_M_ parameter were found, the values of this parameter were always at a high level (above 0.7) in all species. The Area, which is the parameter calculated from OJIP curves (see Table S4), was significantly lower for *Cet* compared to other species (Table S6). The performance index PI_ABS_ (see Table S4) turned out to be highly species dependent. The values determined for *Hyp* was almost twice as high as that for *Cet* (Table S6). There were no significant differences between the 4-min and 15-min dark-adaptation procedure for the values F_O_ and F_M_ within one species (Table S5).

### Adaptation of lichens to changing light conditions

**Fig. 2:**
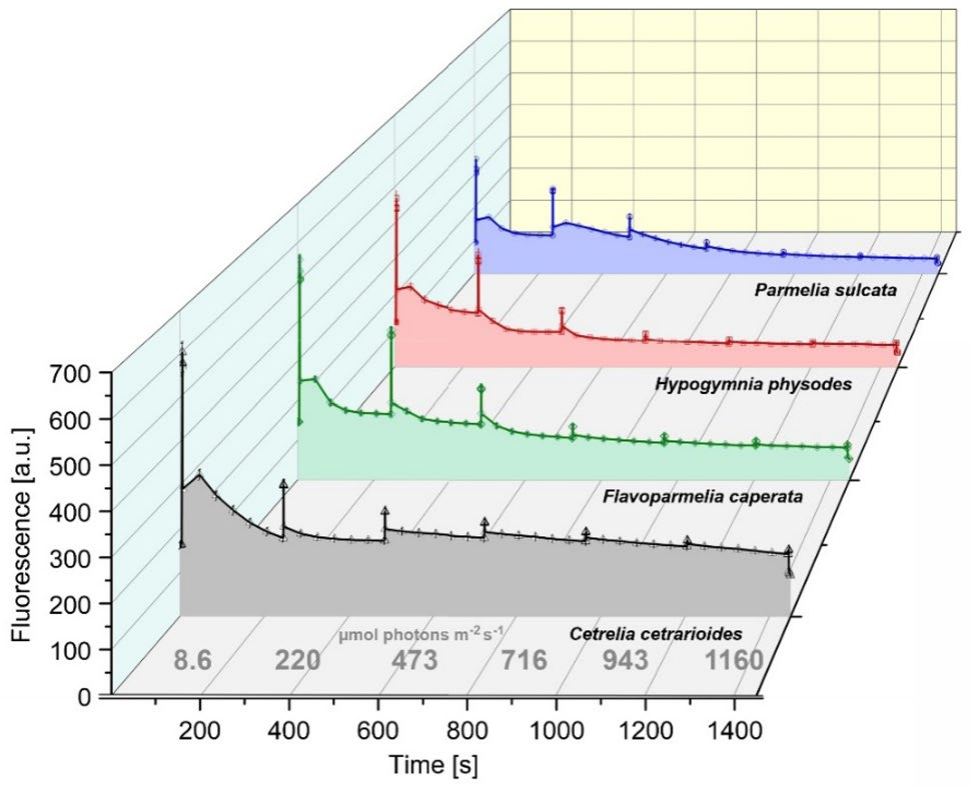
Fluorescence intensity measured under actinic white light (according to the “Light Curve” protocol, see Materials and Methods); the light intensity was changed after each saturating flash applied at regular intervals, as shown along the horizontal axis. The curves are plotted based on the averaged data points (n = 10). Parameters calculated from these curves are shown in Fig. 3

The fluorescence response of dark-adapted lichens to increasing light intensity is shown in Fig. 2. In general, fluorescence signal decreased with the increase of light intensity. However, both the fluorescence level and the shape of curves differed between species.

**Fig. 3:**
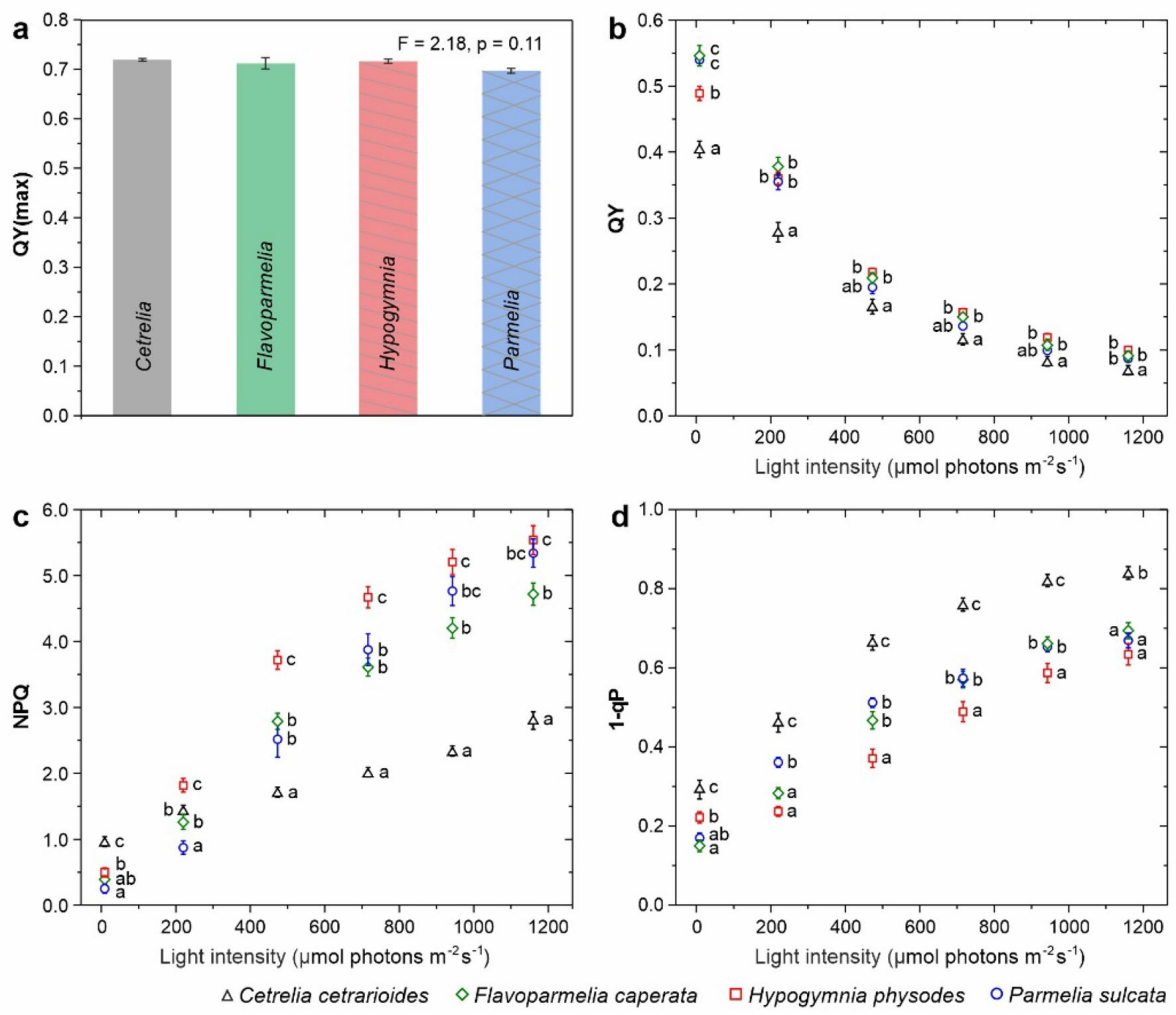
Fluorescence parameters (mean ± SE; n = 10) for particular lichen species calculated from the fluorescence curves (Fig. 2) measured for white actinic light (8–1160 µmol photons m^−2^ s^−1^): QY(max), including results of one-way ANOVA **(a)**, QY **(b)**, NPQ **(c)** and 1-qP **(d)**; the results of Tukey’s (HSD) post-hoc test performed in groups of data from particular light intensities are also provided, various letters indicate statistically significant differences (p < 0.05)

Both the value of F_O_ in dark-adapted lichens and the overall fluorescence level were the highest for *Cet* (Fig. 2). The QY(max) parameter did not differentiate the investigated species and always achieved the values above 0.7 (Fig. 3a). The species showed different kinetic of the adaptation to the increasing light intensity, which was reflected by the values of QY, NPQ, 1-qP parameters (Fig. 3; see Table S4 for an explanation of the parameters). These values were significantly affected by lichen species and light intensity, there were also significant interactions between those variables (Table 1). Nevertheless, the mean QY values for *Cet* were always the lowest compared to other species (Fig. 3b), especially at low light intensity. In line with this, 1-qP for *Cet* reached the highest values throughout the entire spectrum of light intensity, ranging from 0.3 to 0.85 at low and high light intensity, respectively (Fig. 3d). In case of *Hyp*, 1-qP was about 0.2 at light intensity below 200 µmol photons m^−2^ s^−1^ and slowly increased to 0.6 at the highest light intensity (Fig. 3d). The values of 1-qP for *Fla* and *Par* were even lower than for *Hyp* at low light intensity and they were between the values determined for *Cet* and *Hyp* at high light intensity (Fig. 3d).

**Table 1:** Results of two-way ANOVA (p < 0.05) for the effects of lichen species (Species), intensity of white actinic light (Light) and their interaction on the value of QY, NPQ and 1-qP parameters (shown in Fig. 3)

	Variables	SS (Sum of Squares)	MS (Mean Square)	DF (Degrees of Freedom)	η^2^	F	p
QY	Species	0.15	0.05	3	0.49	**70.70**	** < 0.001**
	Light	5.17	1.03	5	0.97	**1473.17**	** < 0.001**
	Species × Light	0.08	0.01	15	0.36	**7.99**	** < 0.001**
	Error	0.15	0.0007	216			
NPQ	Species	90.43	30.14	3	0.66	**143.47**	** < 0.001**
	Light	513.73	102.75	5	0.91	**489.04**	** < 0.001**
	Species × Light	71.51	4.77	15	0.61	**22.69**	** < 0.001**
	Error	45.38	0.21	216			
1-qP	Species	1.53	0.51	3	0.68	**153.67**	** < 0.001**
	Light	7.88	1.57	5	0.92	**474.85**	** < 0.001**
	Species × Light	0.22	0.01	15	0.23	**4.38**	** < 0.001**
	Error	0.72	0.003	216			

The effects in bold are statistically significant

In the studied range of actinic light intensity, non-photochemical quenching (NPQ) increased 2.5 times for *Cet*, 11*–*12 times for *Fla* and *Hyp*, and 21 times for *Par* (Fig. 3c). It should be noted that relatively high values of NPQ were already observed in *Cet* at the low light intensity (8 µmol photons m^−2^ s^−1^) (Fig. 3c).

A similar result was obtained in the analysis performed under red actinic light (Fig. S2, see also Table S7). However, at light intensity lower than 50 µmol photons m^−2^ s^−1^, the values of QY and 1-qP parameters for *Cet* did not always differ from those of other lichens. At higher light intensities, the differences became more pronounced (Fig. S2b and d). It should be noting that excitation pressure (1-qP) for *Hyp* and *Par* was usually below the level of 0.25 (Fig. S2d). The range of changes in the values of NPQ under actinic red light were the narrowest for *Cet* and it is an analogous result to that obtained under white light (Figs. S2c and 3c). Among all investigated species, the values of NPQ in *Cet* were the highest at low light intensity, on the other hand they were relatively low at high light intensity (Fig. S2c).

### Photosynthesis in low and high light intensity – in vivo chlorophyll fluorescence

**Fig. 4:**
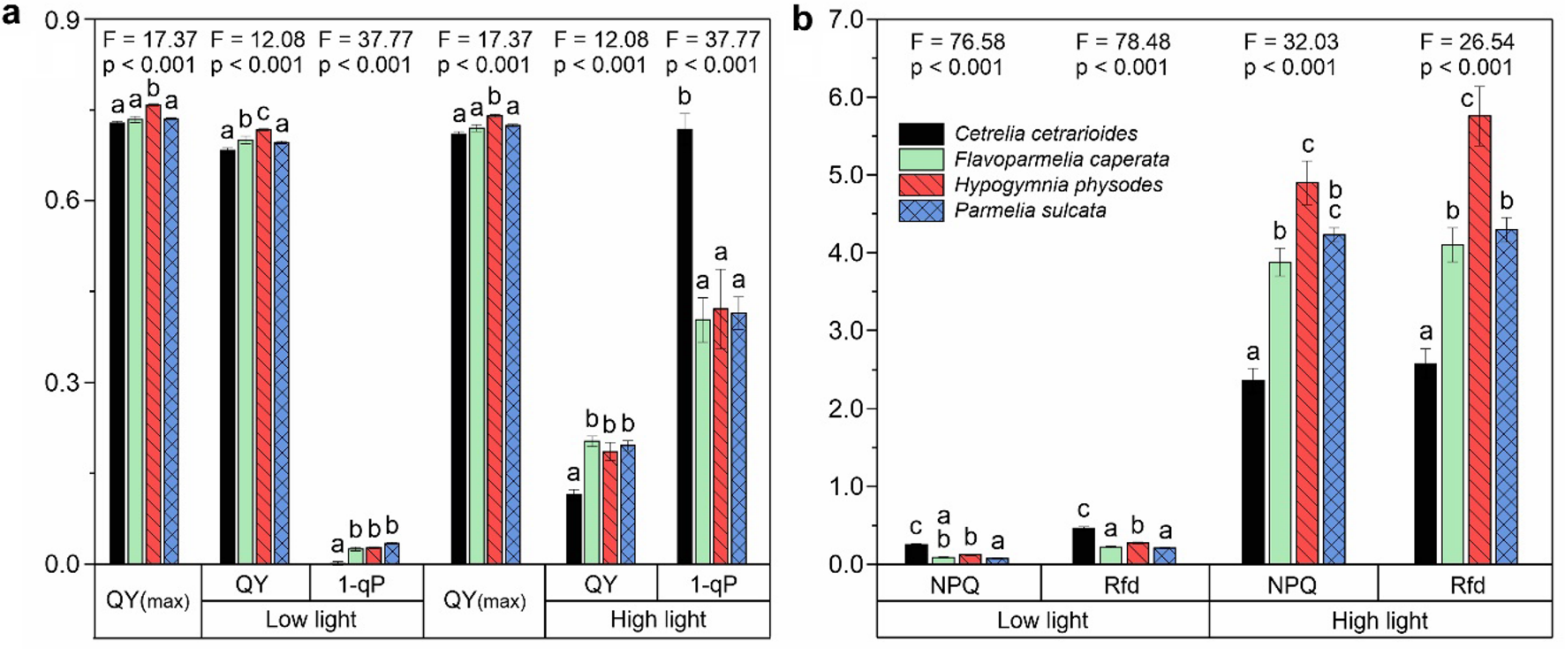
Fluorescence parameters calculated for fluorescence quenching experiments performed under actinic light intensities of 8 µmol photons m^−2^ s^−1^ (low light) and 500 µmol photons m^−2^ s^−1^ (high light). Left panel **(a)** includes parameters with values ranging from 0 to 1, right panel **(b)** values ranging from 0 to 7 (mean ± SE; n = 10); the results of one-way ANOVA (F and p values) are provided, various letters indicate statistically significant differences (p < 0.05) according to the results of Tukey’s (HSD) post-hoc test

In this experiment, performed according to “Quenching Protocol”, we examined the adaptation of photosynthetic activity to a constant low and high light intensities (8 or 500 µmol photons m^−2^ s^−1^). The calculated fluorescence parameters are presented on Fig. 4.

QY(max), i.e*.* maximum PS II quantum yield in dark adapted state, was in the range of 0.71*–*0.76 for examined lichens; the highest values were recorded for *Hyp* both in low light and high light experiment (Fig. 4a). At low light, all species maintained almost maximal value of QY and the deviation was not higher than 6% of QY(max). The highest values of QY were noted for *Hyp* and the lowest for *Cet* and *Par*. This correlated with the values of 1-qP that were less than 0.04 and indicated negligible excitation pressure. At the same time, the excitation pressure was the lowest in *Cet* (Fig. 4a). On the other hand, the values of NPQ for this lichen were much higher than those recorded for other species. The fluorescence decline ratio (R_fd_) was also the highest for *Cet* (Fig. 4b).

At high actinic light, the values of QY parameter were much lower than QY(max) for all studied species and they were the lowest for *Cet* (Fig. 4a). Additionally, the values of 1-qP for *Cet* were almost twice as high as for other species. On the contrary, NPQ values for this species were the lowest (Fig. 4a). Considering NPQ parameter, some significant differences between *Hyp*, *Fla* and *Par* at high light treatment were noted (Fig. 4b). The values of R_fd_ parameter for *Hyp* were the highest and they were even more than twice as high as the values determined for *Cet*. The values of this parameter for *Fla* and *Par* ranked between those recorded for *Cet* and *Hyp* (Fig. 4b).

**Fig. 5:**
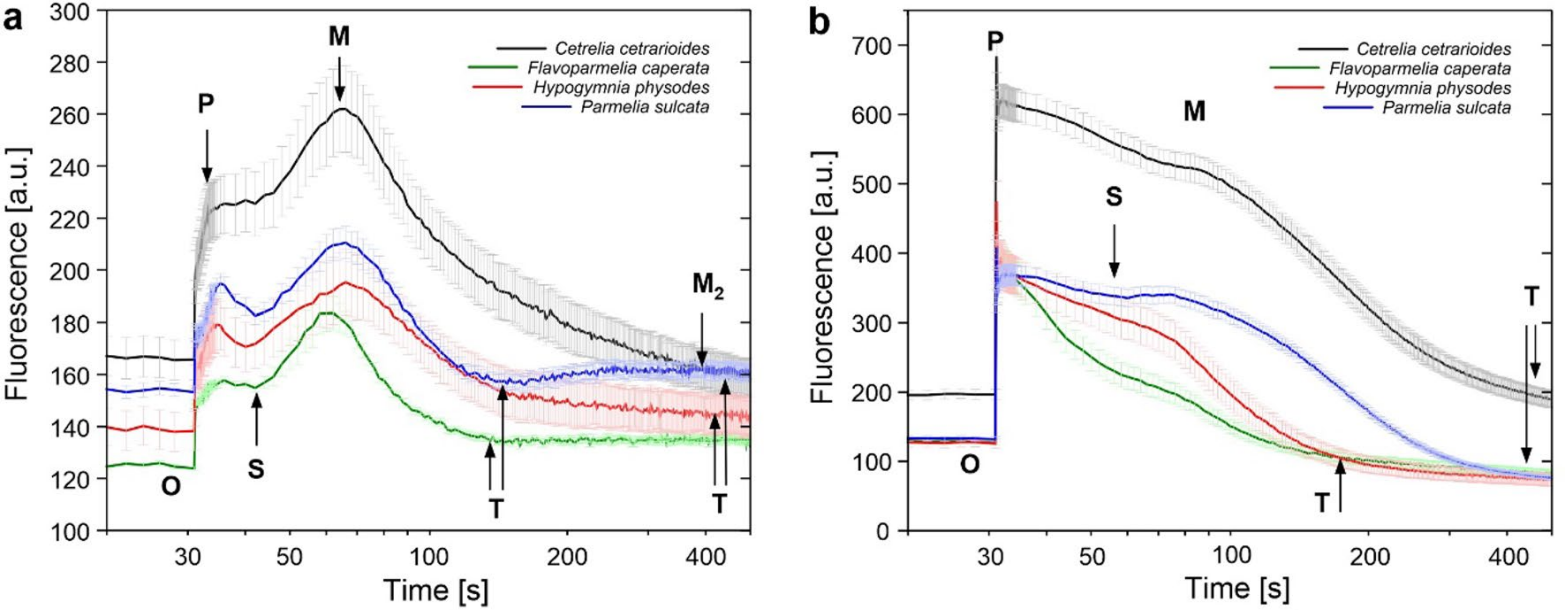
Slow chlorophyll fluorescence transient for particular lichen species recorded at low (8 µmol photons m^−2^ s^−1^) **(a)** and high (500 µmol photons m^−2^ s^−1^) **(b)** intensity of actinic light (the averaged curves, n = 10; SE are marked). The curves are plotted on a logarithmic time scale. The points O, P, S, M, T are indicated on the graphs

Kautsky curves (reflecting chlorophyll fluorescence induction) in continuous light on a minute time scale included an initial fast fluorescence rise (O-P), followed by a slow decline to the steady-state value (T), in some cases via a transient minimum (S) and maximum (M). The slow fluorescence decay proved that the curves represented the so-called PSMT (*Peak, Semi-steady state, Maximum, Terminal state*) curve. The curves (Fig. 5) were recorded both at low and high intensity of actinic light (part of the quenching experiment). PSMT points at low light intensity of actinic light were clearly visible (Fig. 5a). The levels of fluorescence at M point were higher than that observed at P point. Considerably higher fluorescence intensity, especially at the beginning of measurements, was observed for *Cet*. PSMT curves for *Hyp* and *Cet* clearly differed from those obtained for *Fla* and *Par*; for the latter, the second wave (denoted as M_2_) appeared (Fig. 5a). The ratios calculated based on PSMT point values (Table S8) provide more details about the shape and characteristics of the curves. Importantly, the value of M/T ratio for *Cet* was the highest. The time of reaching P point was shorter in case of *Cet* and *Hyp* (5.3*–*5.6 s) than in case of *Par* and *Fla* (about 7 s). Whereas, the time of reaching M point was 37 s, except for *Fla*, for which it was 31 s. As with low light intensity, the level of fluorescence at high light intensity was highest for *Cet* (Fig. 5b). Fluorescence kinetic curves measured at high light were characterised by less pronounced M wave and the fluorescence intensities at M point were significantly lower than at P point (Fig. 5a vs. Figure 5b). The time of reaching M point was between 37 and 55 s, which means it was longer than at low light. The time and the relative fluorescence intensity differed among the species (Fig. 5b). The shortest time was observed for *Hyp* while the longest for *Cet*. Similar time (about 44 s) was noted for *Fla* and *Par*. In the case of *Fla* and *Hyp*, M wave was slightly visible and the steady-state T level was reached much faster as compared to *Par* and *Cet* (Fig. 5b). Similarly, relatively high P/S and P/M ratios were calculated for first two species. The highest M/T ratio, reflecting the greatest decline in fluorescence during adaptation to light, was noted for *Par* (Table S8). It turned out that T level was lower than the initial O level (F_O_) for *Fla*, *Hyp* and *Par* at high light intensity. The effect for *Par* was the most pronounced, a slightly less visible effect was observed for *Fla* and *Hyp* (Fig. 5b).

### Photosynthesis in low and high light intensity – CO_2_ assimilation

**Fig. 6:**
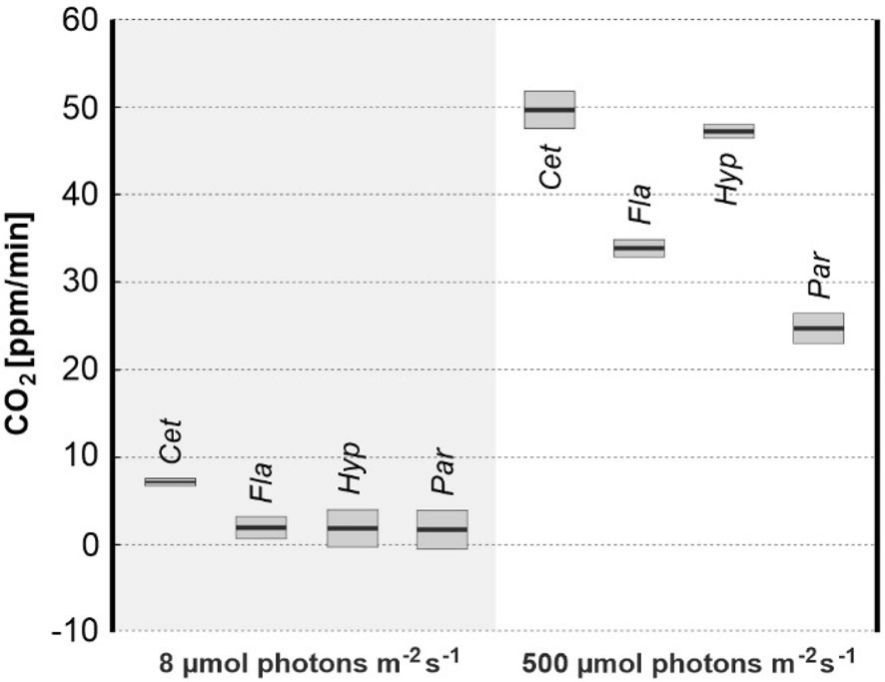
The average rate of CO_2_ assimilation corresponding to gross photosynthesis determined for particular lichen species (mean ± SE; n = 3) measured for 36 cm^2^ area of lichen thalli; for species abbreviations, see Materials and Methods

The average gross CO_2_ assimilation for each lichen species calculated as the difference between the rates of net photosynthesis and dark respiration is shown in Fig. 6. These results provide information on the photosynthetic activity of the photobionts, including light and dark photosynthetic reactions. The rate of CO_2_ assimilation measured at high light intensity was much higher than at low light for all lichen species. At low light, the photosynthesis rate for *Cet* was considerably higher than that observed for other species. In contrast to *Cet*, the obtained values for *Fla*, *Hyp* and *Par* were close to or slightly higher than zero. This means that *Cet* demonstrated the highest photosynthetic activity under low light conditions. Noticeable differences in the rate of CO_2_ assimilation between species were noted at high light intensity. The values recorded for *Cet* and *Hyp* were higher than for *Fla*, while the rate of CO_2_ assimilation was apparently the lowest in case of *Par*.

### Low temperature fluorescence spectra

**Fig. 7:**
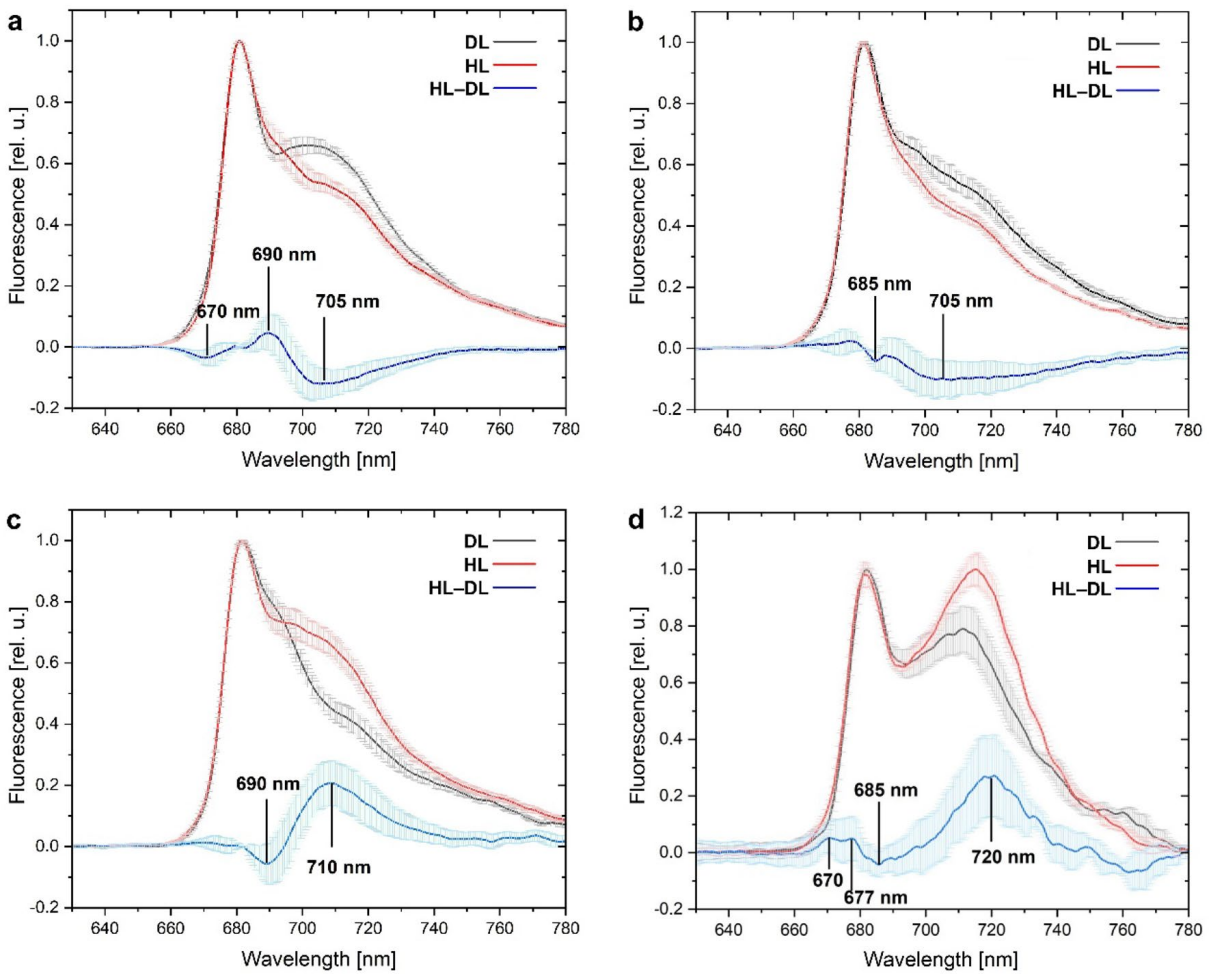
Fluorescence spectra at 77 K for lichen thalli homogenates prepared after 30 min incubation in dim light (DL, < 5 µmol photons m^−2^ s^−1^) and high light (HL, 500 µmol photons m^−2^ s^−1^); excitation wavelength = 435 nm. HL-DL is a differential spectrum calculated by a subtraction of HL and DL spectra. Lichen species: *Cetrelia cetrarioides* **(a)**, *Flavoparmelia caperata* **(b)**, *Hypogymnia physodes* **(c)**, *Parmelia sulcata* **(d)**

Complex fluorescence emission spectra were observed with the maximum at 681 nm, and a shoulder or a second maximum at the right-site band. The latter was affected by illumination, nevertheless the effect was clearly species-dependent. For *Cet* and *Fla,* the relative fluorescence at 705 nm decreased due to treatment with high light (500 µmol photons m^−2^ s^−1^ for 30 min; Fig. 7a and b). The opposite effect was noted for *Par* and *Hyp.* High light treatment resulted in the increase of relative fluorescence in the range of 710*–*720 nm for these species (Fig. 7c and d). Moreover, a very clear second maximum was observed at 715 nm for *Par*. On the other hand, the decrease of fluorescence bands at 685*–*690 nm was observed (Fig. 7c and d).

## Discussion

Widespread and often abundant occurrence is the domain of numerous generalist lichens or those that prefer open areas (Liška and Herben 2008). On the other hand, many epiphytic lichens with narrow ecological tolerance have a very limited distribution range and found shelter only in the interior of close-to-natural deciduous and mixed forests (Nordén et al. 2007, 2014). The recognition of tolerance limits of lichens and identifying factors that may affect their distribution seem to be an urgent matter in the light of the reports on dramatic losses in the diversity in temperate deciduous forests over the last century, especially in the face of climate change (see Hauck et al. 2013). Strong attachment to forests results mainly from their habitat specificity; the interior of a large forest provides shade, slows down the wind, maintains high humidity, and tempers fluctuations in temperature (Chen et al. 1999). In contrast, trees of non-forested areas constitute a habitat that is highly variable and extreme in terms of climatic conditions (Chen et al. 1993). There is evidence to support that the occurrence of stenoecious forest lichens is controlled by a delicate balance between their susceptibility to light stress, essential light availability and a complex of more or less directly related microclimatic factors nested in the forest habitat (Gauslaa and Solhaug 1996; Gauslaa et al. 2006, 2012; Heber et al. 2000). To better understand the actual effect of light factor on the distribution and habitat requirements of lichens, we selected species with different ecological properties for the study, i.e. *Cet* which prefers shadowed sites (see Table S3) and is treated as old-growth forest indicator (Table S1) and *Fla*, *Hyp* and *Par* that are not directly associated with forest habitat, do not avoid light, though show a rather different photophilicity.

Energy absorbed by the accumulated chlorophyll or carotenoid molecules is used to power photochemical reactions in PSII/PSI photosystems, or dissipated as heat, or emitted as fluorescence (Murchie and Lawson 2013; Stirbet et al. 2020). These processes are competitive and the analysis of chlorophyll fluorescence allows to monitor both the photochemical reactions and photosynthetic electron transport, as well as the energy dissipated (Roháček et al. 2008). Chlorophyll fluorescence analyses can be supplemented by measurements of the rate of CO_2_ assimilation, which provide information on the intensity of photosynthetic dark reactions (i.e. the Calvin-Benson cycle). This reaction cycle is in turn strongly dependent on light photosynthetic reactions as it uses their products, i.e. NADPH and ATP. Important aspects of the functioning of the photosynthetic apparatus in the studied lichens are summarized below.

### Photosystem II properties in dark adapted lichens

Maximal quantum yield of PSII, determined by both pulse-modulation and continuous excitation fluorimeter, varied between species to small extent (Figs. 3a, 4 and S2a, Table S6), and reached the highest level previously reported for chloro-lichens (Jensen 2002; Piccotto and Tretiach 2010). This means that, regardless of the species, photosynthetic activity with maximal efficiency characterized the examined thalli.

The highest level of overall fluorescence is the most apparent trait that distinguishes *Cet* from other lichens and points to its peculiarity (Figs. 1, 2 and 5, see also Table S5). The OJIP curve for *Cet* differs from the others mainly due to an increased K level and a relatively flat I-P phase (Fig. 1b). The noticeable K band and, in consequence, the highest ΔV_OJ_ and V_K_/V_J_ values (Fig. 1b) probably indicate some defects at the donor site of PSII. This may be related to the impairment of oxygen evolving complex and inhibition of donating electrons to PSII due to heat/drought and high-light stresses (Strasser et al. 2004). The K step was also observed under natural conditions and explained by changes in the architecture of the PSII antenna (Srivastava et al. 1997). The K band was already observed in Antarctic lichens at high temperature (Bednaříková et al. 2020).

The I-P phase reflects the redox state of the plastoquinone (PQ) pool and is a consequence of the reduction of PQ with electrons from PSII and PQ reoxidation due to the ongoing electron transport by PSI (Lazár, 2006). The suppressed I-P phase and simultaneously very low value of ΔV_IP_ was observed in *Cet* (Fig. 1b). Some deficiency in the water splitting complex performance as well as relatively small pool of PQ may be peculiarity of *Cet* (see Table S6 – Area). In contrast to *Cet*, a clear pits separating the I and P points were observed, and consequently, significantly higher values of ΔV_IP_ were calculated for other investigated species, especially in case of *Par* (Fig. 1b). It has been suggested that pits reflect oxidation of the PQ pool due to the PSI activity, whereas the following rise in the I-P phase reflects the reduction of PSI reaction center and its acceptors (Lazár 2006). Based on the results presented here it can be concluded that the properties of the dark-adapted PSII and kinetic of the linear electron transport clearly distinguish *Cet* from the generalist lichens. Further experiments are required to explain in detail mechanisms activated in *Cet*.

We observed that the level of F_O_ for *Cet* was always higher than that noted for other species (Figs. 1, 5, Table S5). At the same time, the direct relation between the values of the F_O_ parameter and the content of chlorophyll *a* or the total chlorophyll was not found when comparing the studied lichen species (Fig. 1 and Table S5 vs. Table S2). Given the discussion by Kalaji et al. (2014), the correlation between the chlorophyll content in leaves and fluorescence level is not obvious. Moreover, interspecies comparison for lichens may be problematic. The share of fungal hyphae to the total weight of the thallus can vary greatly between species which has a direct impact on the chlorophyll content determined per thallus dry weight. Nevertheless, we noticed some relation between the level of the Fo parameter and the Chl a/b ratio (compare Fig. 1 and Table S5 with Table S2). This ratio depends on the relative amount and/or composition of the photosynthetic antennae as well as photosystems which may impact the fluorescence quantum yield of chlorophyll and explain differences in the observed fluorescence level (see Kalaji et al. (2017) for discussion).

### Photosynthetic activity under continuous light

The functioning of PSII was studied both in response to stepwise increases of light intensity (Figs. 3 and S2) as well as in constant light of low or high intensities (Figs. 4 and 5). The results vary between species and reveal their different kinetic of adaptation to light, especially when ecological group generally is taken into account. This is further emphasized by the fact that at the level of a given species, the results of both experiments were very consistent.

The values of QY for *Cet* were always the lowest among the examined species (Figs. 3b, S2b and 4a), which indicates a relatively low PSII operating efficiency in the light adapted state (i.e. low effective PSII quantum yield induced in light) (Genty et al. 1989; Maxwell and Johnson 2000). On the other hand, the values of 1-qP for *Cet* were the highest (Fig. 3d). The 1-qP parameter (excitation pressure on PSII) is used to denote the proportion of reduced PSII reaction centers (i.e. closed PSII) (Maxwell and Johnson 2000; Roháček et al. 2008). Therefore, relatively high proportion of PSII reaction centres in *Cet* is reduced probably due to the lack of oxidized PSII acceptors and discontinue the electron transport. PSII limitation at the acceptor site is associated with small PQ pool (see Table S6 – the smallest Area for *Cet*). High excitonic pressure is especially noticeable for *Cet* tested under high light intensity, where the 1-qP value was almost twice as high as for other species (Fig. 4a). Importantly, the electron transport via PSII turned out to be efficient in the thalli of *Cet* adapted to low light intensity, where the 1-qP value was close to zero, which points to negligible fraction of reduced PSII (Fig. 4a). All these facts prove that *Cet* performs very efficiently in low light conditions but, unlike the other species, deficiently at high light. This is fully in line with the no-compromise habitat requirements of this species, the interior of an ancient forest creates a chance for this lichen to exist. This may be also interpreted as a symptom of specific adaptation of *Cet* to low light condition.

The highest QY values were recorded for *Hyp*, especially under high light conditions (Figs. 3b, S2b and 4a). On the other hand, the values of 1-qP were the lowest among the studied lichens (Figs. 3d, S2d and 4a). Moreover, the change span in values of QY across the light intensities was relatively the smallest. Almost 50% of PSII reaction centres is oxidized (i.e. open PSII) even at the highest light intensity pointing to the efficient PSII photochemistry (Genty et al. 1990). This proves the functional plasticity of PSII and good adaptation of *Hyp* to a wide range of light intensity. Indeed, this species is non-specific in terms of habitat type and can be easily found inside and outside the forest (e.g. Kubiak and Osyczka 2020); besides, in fact, it is one of the most common ubiquitous foliose lichen.

Similar observations regarding PSII efficiency at high light intensity were made for *Fla* and *Par* (Figs. 3b, S2b and 4a). However, the values of 1-qP tended to be higher than those obtained for *Hyp,* indicating relatively high proportion of reduced PSII reaction centres. Differences were observed at low light intensity where PSII operating efficiency (QY) was higher whereas the excitation pressure (1-qP) was lower for *Fla* and *Par* as compared to *Hyp*. Thus at low light, PSII units are mostly in the open state and primary reactions of photosynthesis are not inhibited in case of first two species. All these characteristics fit very well especially with the distribution pattern of *Fla*, as it often appear in less dense gaps of old forests or forest edges and on ‘veteran’ trees that provide quite a lot of shade (e.g. Kościelniak 2013).

The slow Kautsky kinetic (PSMT curve) discloses combined effect of the photochemical and non-photochemical quenching (Stirbet and Govindjee 2016) related to the photosynthetic activity and dissipation of excess of absorbed energy in the form of heat, respectively. Moreover, carbon assimilation during the Calvin-Benson cycle and chlororespiration also modulate chlorophyll fluorescence on a minute time scale and affect the appearance of the transitional maximum M (Papageorgiou et al. 2007; Stirbet and Govindjee 2016). There is no universal standard for the character of PSMT curve, it represents both the nature and history of particular photosynthetic organisms. In general, the S-M phase is observed in vascular plants mostly under low light conditions (Strasser et al. 1995), while in cyanobacteria the M peak is the dominant, although observed much later than in plants (Tsimilli-Michael et al. 2009). We observed species specific shape of curves both at low and high light intensity (Fig. 5). At high light, curves were more similar to those of plants and differed one from the other especially in terms of intensity and duration of the S-M phase; this wave was most visible for *Par* (Fig. 5b, Table S8). At low light, the S-M phase was significantly higher than the P level (Fig. 5a, Table S8). A clear increase in chlorophyll fluorescence to the M level reflects the consumption of ATP and NADPH during carbon reduction and depends on CO_2_ supply. In case of lichens, a certain limitation of CO_2_ diffusion through the thallus layers may occur. The studied lichens form a cortex layer of similar thickness (Table S1); however, the structure of fungal filaments in particular layers may vary between species. It may be also that the light-regulated activation of the Calvin cycle responses is weakened under low light conditions. Similar shape of the PSMT curves (Fig. 5a) were observed for lichens in response to decrease in temperature of thallus (freezing stress) (Marečková and Barták 2016; Mishra et al. 2015). Conti et al. (2014) observed the level of M peak higher than P for *S. vesuvianum* and interpreted this fact as originating either from less effective reoxidation of PQ pool or as contribution of F_O_ (during actinic light) to the overall chlorophyll fluorescence signal.

Measurements of gross CO_2_ assimilation (Fig. 6) confirmed fluorescence experiments and revealed that under low light the photosynthetic activity of *Cet* is much higher than in other lichen species. Under high light, *Cet* also efficiently assimilates CO_2_, even though the fluorescence analysis revealed high excitation pressure (1-qP) and low QY values (Fig. 4) which points to the saturation of the electron transport chain. However, it should be emphasized that the relationship between the photosynthetic electron transport rate, related to the QY value, and the assimilation of CO_2_ is complex. This is due to the existence of electron sinks in chloroplasts, including chlororespiration (see Kalaji et al. 2017 for discussion).

### Functioning of photosynthetic antennae – non photochemical quenching

Pigments of photosynthetic antennae absorb light and transfer the excitation energy to PSII and PSI reaction centers, providing energy to power the electron transport (Croce and van Amerongen 2014). In addition, the harmless dissipation excess excitation energy in the form of heat protects the photosynthetic antennae. This can be monitored by analyzing the value of the NPQ parameter (Goss and Lepetit 2015; Sohbat 2022). A wider range of changes in the values of NPQ indicates a greater ability of antennae to dissipate excess excitation energy. Then, the photosynthetic apparatus is able to smoothly regulate the consumption and dissipation of excitation energy and to adapt to changes in light intensity.

There is a significant difference in the NPQ value between *Cet* and the rest of species. Even at low light intensity, NPQ in *Cet* is already relatively high (Figs. 3c, S2c and 4a). Moreover, for increasing light intensity, NPQ in *Cet* showed the smallest range of changes. Consequently, under high light, excess energy is transferred to PSII reaction centers and contributes to the observed relatively high excitonic pressure (Figs. 3d, S2d and 4a). In contrast to *Cet,* the largest NPQ range was recorded for *Par* (Figs. 3d, S2d and 4a). Thus, this species is able to adjust the proportion of light dissipation to the ambient conditions. Indeed, among the studied species, *Par* can be considered the most photophilous and associated with open areas.

Non photochemical quenching contribute to the PSMT curve (Papageorgiou et al. 2007). It has been suggested that the increase in chlorophyll fluorescence in the S-M phase is a signature for the transition from state 2 to state 1 in green algae *C. reinhardtii* (Kodru et al. 2015). In turn, the P-S and M-T phases were attributed to the energy-dependent component of NPQ. State transition balance absorbed energy between photosystems (PSI and PSII) by mobilizing specific light-harvesting complex (LHCII antenna) (Minagawa 2013). The state 2 relates to the situation when the antennae are bound to PSI, whereas the state 1 occurs when they are bound to PSII. According to the interpretation of Kodru et al. (2015), transition from state 2 to state 1 was observed in our study for lichens under low actinic light (Fig. 5a). This observation is further supported by 77 K fluorescence spectra (Fig. 7). For all species, the fluorescence band with a maximum at 681 nm dominated over the bands between 710 and 730 nm. These bands originate from the PSII and PSI complexes, respectively (Lamb et al. 2018). For *Hyp* and *Par,* the high light treatment resulted in the relative increase of fluorescence intensity between 710 and 720 nm (Fig. 7c and d; see the HL-DL spectra) which can be attributed to core PSI complex (Andreeva et al. 2003; Yamamoto et al. 2013). In addition, the decrease of the bands at 690 nm (*Hyp)*/685 nm (*Par*) was observed. They were attributed to PSII reaction center/core antennae (Andrizhiyevskaya et al. 2005; Yamamoto et al. 2013). These spectral changes point to state 1 to state 2 transition in *Hyp* and *Par* induced by high light treatment (Kruse et al. 1999; Węgrzyn et al. 2022). In contrast, spectral changes induced by high light treatment in *Cet* included the increase of the band at 690 and the decrease of the band at 705 nm (Fig. 7a; see the HL-DL spectra). The enhancement of the PSII fluorescence, observed at 690 nm, proves that more excitation energy is trapped in the intrinsic CP antenna (CP43 and CP47) (Andrizhiyevskaya et al. 2005; Yamamoto et al. 2013). This is probably due to disaggregation of the LHCII trimers (fluorescent at 700 nm) and/or more efficient energy coupling with the intrinsic antennae (Andreeva et al. 2003). This is in line with the observed increase of the excitonic pressure on PSII (Fig. 5a, see 1-qP parameter). The effect of high light on *Fla* was similar to that observed for *Cet*, but much less pronounced (Fig. 7b). It is evident that the ability to use of light efficiently by scattering of excess energy and/or its redistributing between photosystems is specific for generalist lichens.

## Conclusions

**Fig. 8:**
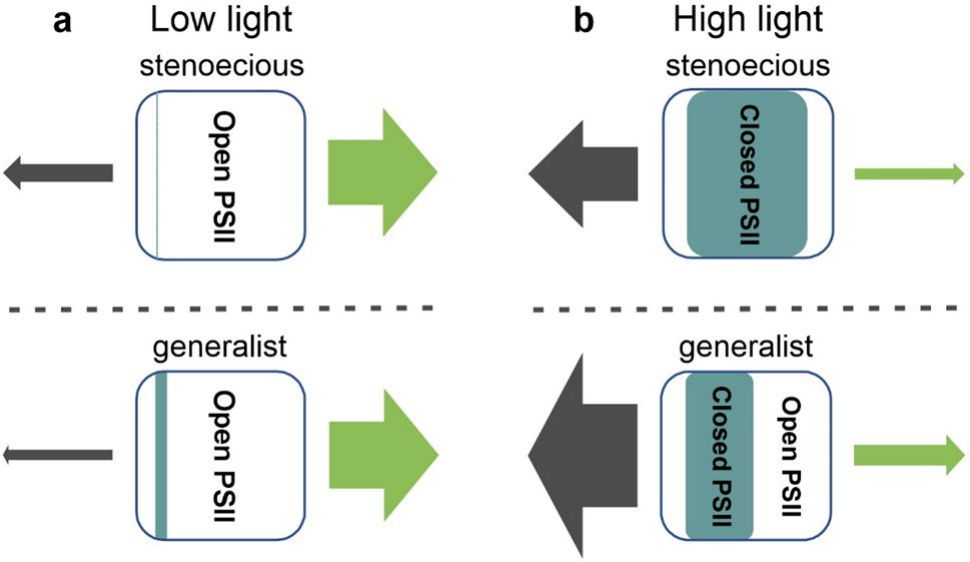
Simplified model of energy flow related to the photobiont photosynthetic activity in stenoecius and generalist lichens under low **(a)** and high light **(b)** conditions. The green and gray arrows represent energy used for photosynthesis (photochemical quenching) and energy dissipated (non-photochemical quenching), respectively. The thickness of the arrows shows the relative energy. The proportion between open (white) and closed (colored area) reaction centers is also shown

The results of our experiments clearly proved that light conditions constitute a key factor for the lichen photosynthetic performance and the functional plasticity of the photosynthetic apparatus of algal component in relation to changing light conditions differs considerably between species. The difference in the functioning of the photosynthetic apparatus in low and high light between stenoecious lichen and generalist lichen is schematically illustrated in Fig. 8. The old-growth forest lichen *Cet* demonstrates definitely lower range of energy dissipation than other species, although it assimilates CO_2_ efficiently both at low and high light. Distribution of the excitation energy in the case of *Fla* and *Hyp* is similar to that in *Par*; however, the last dissipates the excess energy most efficiently. Disclosed differences may be of great importance for the distribution of lichens and persistence of stenoecious species in the environment. For example, a seemingly insignificant change in the structure of a forest stand (e.g. selective logging, alteration of tree species composition) may modify the light conditions and, consequently, pose a threat to a sensitive species and, on the other hand, promote development of generalist lichens.

## Supplementary Information

Below is the link to the electronic supplementary material.

Supplementary file1 (PDF 656 KB)

## Data Availability

Data are presented in this published article and its Supplementary Information. Original data are available from the corresponding authors on reasonable request.
