# Supplementary material for: The pattern of photosynthetic response and adaptation to changing light conditions in lichens is linked to their ecological range
Source: Photosynth Res. 2023 Mar 28;157(1):21–35. doi: 10.1007/s11120-023-01015-z (PMC10282042; doi:10.1007/s11120-023-01015-z)
Supplement: Supplementary file 1 — Supplementary file1 (PDF 656 KB) [file 11120_2023_1015_MOESM1_ESM.pdf]

SUPPLEMENTARY MATERIAL

The pattern of photosynthetic response and adaptation to changing light conditions in lichens is linked to their ecological range

Piotr Osyczka[1], Beata Myśliwa-Kurdziel[2]
[1] Jagiellonian University in Kraków, Faculty of Biology, Institute of Botany, Gronostajowa 3, Kraków 30-387, Poland
[2] Jagiellonian University in Kraków, Faculty of Biochemistry, Biophysics and Biotechnology, Department of Plant Physiology and Biochemistry, Gronostajowa 7, 30-387 Kraków, Poland

Corresponding author: b.mysliwa-kurdziel@uj.edu.pl

Fig. S1 Epiphytic lichens selected for the study (abbreviations used in the main text of the article are given in parentheses).
A – *Cetrelia cetrarioides* (Duby) W.L. Culb. & C.F. Culb. (*Cet*)
B – *Flavoparmelia caperata* (L.) Hale (*Fla*)
C – *Hypogymnia physodes* (L.) Nyl. (*Hyp*)
D – *Parmelia sulcata* Taylor (*Par*)

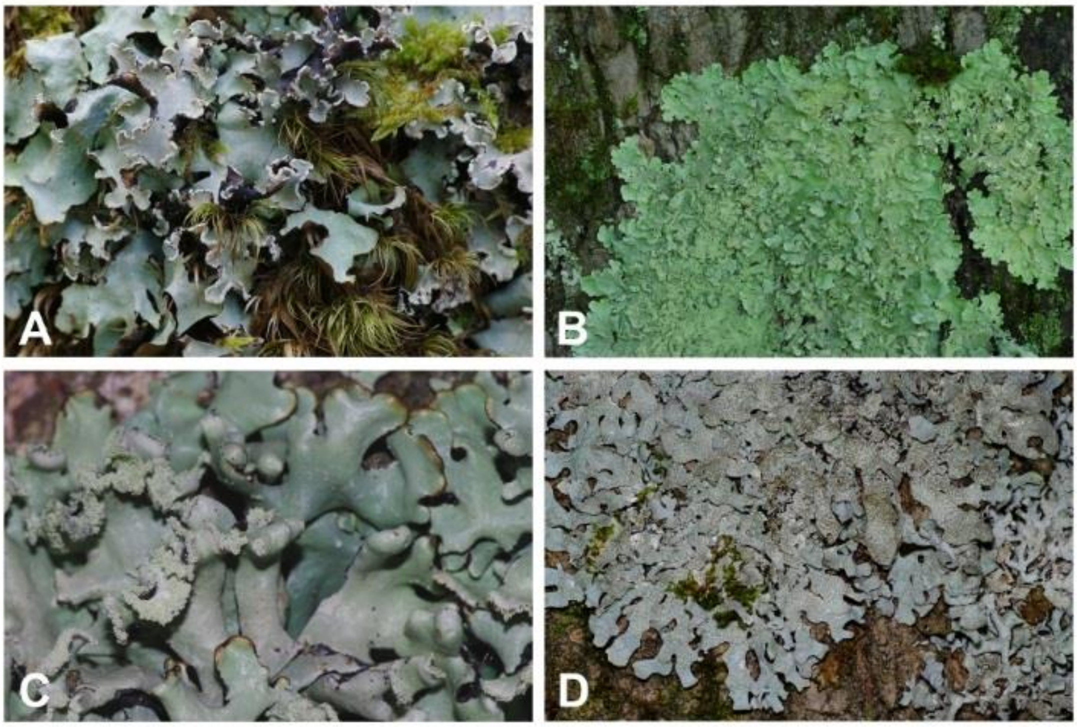

Table S1 Brief characteristics of the studied epiphytic lichens.

Lichen species	Growth form / Reproductive strategy	Algal component	Upper cortex thickness [1]	Algal layer thickness [1]	Indicative value [2] / Light value (L) [3] / Solar irradiation value (S) [4]
Cetrelia cetrarioides (Duby) W.L. Culb. & C.F. Culb.	Foliose (broad lobed) / mainly asexual	Green algae (trebouxioid)	18–29 (22) µm	20–37 (26) µm	Pf, OGf / L 5 / S 3
Flavoparmelia caperata (L.) Hale	Foliose (broad lobed) / mainly asexual	Green algae (trebouxioid)	17–27 (23) µm	20–31 (25) µm	RMf / L 6 / S 3–4
Hypogymnia physodes (L.) Nyl.	Foliose (narrow lobed) / mainly asexual	Green algae (trebouxioid)	19–34 (26) µm	22–38 (29) µm	Df / L-7 / S 3–4
Parmelia sulcata Taylor	Foliose (narrow to broad lobed) / mainly asexual	Green algae (trebouxioid)	19–28 (25) µm	22–33 (27) µm	Mf / L-7 / S 3–5

[1] based on own measurements made for randomly selected thalli fragments of lichen individuals used in this study (10 specimens × 3 cross-section measurements, n = 30); thickness ranges and mean values (in parentheses) are provided
[2] acc. to: Cieśliński 2003; Motiejūnaitė et al. 2004
[3] acc. to: Wirth 2010
[4] acc. to: Nimis 2022
Abbreviations: Pf – primeval forest, OGf – old-growth forest indicator, RMf – regenerating managed forest, Df – degenerated forest, Mf – managed forest

Table S2 Parameters related to the chlorophyll content (mean ± SE, n = 10) determined in the thalli of studied lichen species. Specification based on measurements performed on lichen material collected for this study; for details of the analysis procedure, see Rola et al. 2019. The results of one-way ANOVA (F and p values) are provided; various letters indicate statistically significant differences ($p < 0.05$) according to Tukey's (HSD) post-hoc test.

	**Chl *a* (µg/mg DW)**	**Chl *b* (µg/mg DW)**	**Chl *a*+*b* (µg/mg DW)**	**Chl *a*/*b***
Cetrelia cetrarioides	1.12±0.01 **c**	0.30±0.01 **a**	1.42±0.01 **c**	3.77±0.03 **c**
Flavoparmelia caperata	0.71±0.02 **a**	0.29±0.01 **a**	1.00±0.02 **a**	2.43±0.02 **b**
Hypogymnia physodes	1.35±0.04 **d**	0.67±0.03 **c**	2.02±0.07 **b**	2.03±0.08 **a**
Parmelia sulcata	0.89±0.02 **b**	0.43±0.02 **b**	1.32±0.03 **c**	2.07±0.06 **a**
F value	**128.13**	**88.19**	**118.56**	**227.76**
p value	**< 0.001**	**< 0.001**	**< 0.001**	**< 0.001**

Table S3 Demonstrative light conditions for the examined lichen species measured at their host tree trunks on a clear day (early afternoon hours) in the middle of summer season (n = 50). Measurements were carried out at the same day using Kipp & Zonen PAR Quantum Sensor.

**Lichen**	**Light intensity range**
Cetrelia cetrarioides	5–20 µmol photons m^{-2} s^{-1}
Flavoparmelia caperata	50–430 µmol photons m^{-2} s^{-1}
Hypogymnia physodes	120–500 µmol photons m^{-2} s^{-1}
Parmelia sulcata	100–750 µmol photons m^{-2} s^{-1}

Table S4 Fluorescence parameters analysed in the present study derived from saturating pulse and modulated fluorescence methods (according to: Genty et al. 1989; Horton and Ruban 1992; Horton et al. 1996; Lichtenthaler and Miehe 1997; Paoli et al. 2010; Kalaji et al. 2016).

**Parameter**	**Description**
Saturating puls method (OJIP)	
F_O	Minimum Chl *a* fluorescence yield in the dark-adapted state
F_M	Maximum Chl *a* fluorescence yield in the dark-adapted state
F_V/F_M	Ratio representing the potential quantum yield of primary photochemistry
PI_{ABS}	Performance index, a global indicator that resumes the contribution of all parameters
ΔV_{OJ}	Changes of amplitude of relative variable fluorescence in OJ phase
ΔV_{JI}	Changes of amplitude of relative variable fluorescence in JI phase
ΔV_{IP}	Changes of amplitude of relative variable fluorescence in IP phase
V_K/V_J	Ratio of variable fluorescence in time 0.3 ms to variable fluorescence in time 2 ms as an indicator of the PSII donor side limitation (K-band)
Area	The area above the fluorescence curve between F_O and F_M; proportional to the pool size of the electron acceptors Q_A on the reducing side of PSII.
Modulated fluorescence	
F_O, F_O’	Minimum Chl *a* fluorescence yield in the dark-adapted and light-adapted state, respectively
F_M, F_M’	Maximum Chl *a* fluorescence yield in the dark-adapted and light-adapted state, respectively
QY(max)	Maximum PSII quantum yield in dark-adapted state; F_V/F_M
QY	Effective PSII quantum yield; PSII quantum yield induced in light
NPQ	Non-photochemical quenching
1-qP	Excitation pressure of the PSII reaction center; estimate of the fraction of closed PSII reaction centers $PSII_{closed}$ / ($PSII_{open}$+ $PSII_{closed}$); calculated based on the measured qP coefficient
R_{fd}	Fluorescence decline ratio; empiric parameter used to assess plant vitality

Table S5 The values of fluorescence parameters F_O and F_M determined in particular lichen species. The results of the dependent samples t-test (t and p values; 15 min vs. 4 min dark adaptation) are provided.

	**Parameter**	**Dark adaptation**	**Mean (n=10)**	**SE**	**t**	**df**	**p**
Cetrelia cetrarioides	F_O	15 min	925	42	-1.09	9	0.31
		4 min	938	47			
	F_M	15 min	3798	91	2.08	9	0.07
		4 min	3758	99			
Flavoparmelia caperata	F_O	15 min	721	33	-0.35	9	0.73
		4 min	745	36			
	F_M	15 min	2985	85	0.71	9	0.50
		4 min	2939	84			
Hypogymnia physodes	F_O	15 min	663	27	-0.41	9	0.69
		4 min	684	28			
	F_M	15 min	2306	93	-0.26	9	0.79
		4 min	2348	92			
Parmelia sulcata	F_O	15 min	522	30	-0.48	9	0.67
		4 min	529	43			
	F_M	15 min	2245	188	0.87	9	0.41
		4 min	2191	210			

Table S6 Parameters related to the chlorophyll fluorescence (mean ± SE; n = 10) determined in examined lichens (healthy fully hydrated thalli). The results of one-way ANOVA (F and p values) are provided; various letters indicate statistically significant differences ($p < 0.05$) according to Tukey's (HSD) post-hoc test.

**Lichen**	**Parameter related to photosynthesis efficiency**		
	**F_V/F_M**	**PI_{ABS}**	**Area**
Cetrelia cetrarioides	0.73 ± 0.005 **a**	0.37 ± 0.028 **a**	28846 ± 3129 **a**
Flavoparmelia caperata	0.74 ± 0.013 **ab**	0.67 ± 0.069 **bc**	46169 ± 2994 **b**
Hypogymnia physodes	0.76 ± 0.006 **b**	0.71 ± 0.039 **c**	40237 ± 2432 **b**
Parmelia sulcata	0.73 ± 0.008 **ab**	0.52 ± 0.041 **ab**	39479 ± 1944 **b**
F value	**3.01**	**10.88**	**7.11**
p value	**0.04**	**< 0.001**	**< 0.001**

Fig. S2 Fluorescence parameters (mean ± SE; n = 10) for particular lichen species calculated from the fluorescence light curves measured for red actinic light (20–240 µmol photons m^{-2} s^{-1}): QY(max), including results of one-way ANOVA **(a)**, QY **(b)**, NPQ **(c)** and 1-qP **(d)**; the results of Tukey's (HSD) post-hoc test performed in groups of data from particular light intensities are also provided, various letters indicate statistically significant differences ($p < 0.05$).

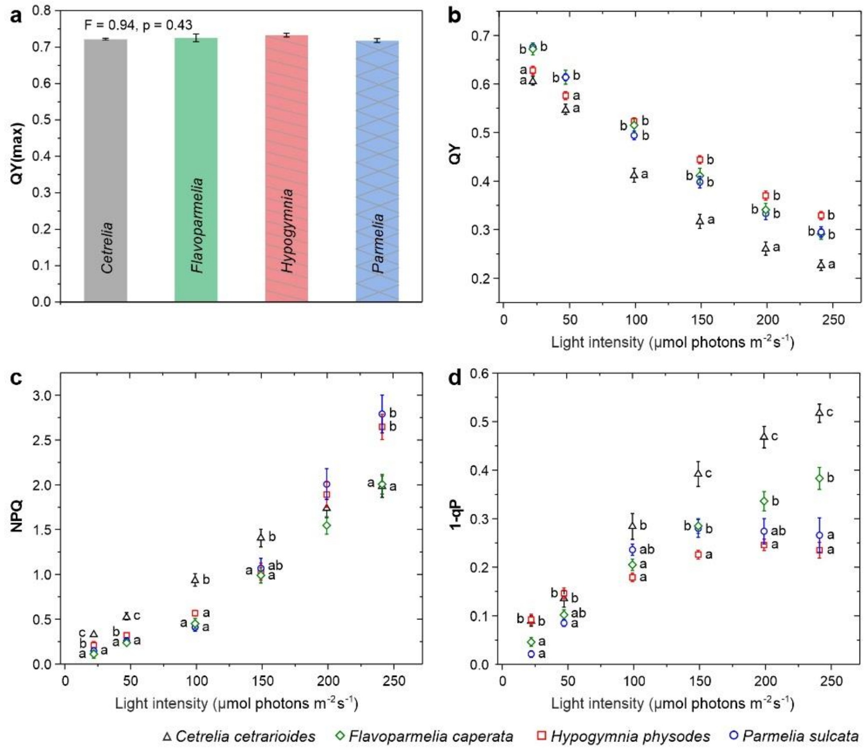

Table S7 The results of two-way ANOVA ($p < 0.05$) for the effects of lichen species (Species), intensity of red actinic light (Light) and their interaction on the value of QY, NPQ and 1-qP parameters, the effects in bold are statistically significant.

	**Variables**	**SS (Sum of Squares)**	**MS (Mean Square)**	**DF (Degrees of Freedom)**	η^2	**F**	**p**
**QY**	Species	0.28	0.09	3	0.53	**81.76**	**< 0.001**
	Light	4.16	0.83	5	0.94	**709.58**	**< 0.001**
	Species × Light	0.07	0.004	15	0.21	**3.95**	**< 0.001**
	Error	0.25	0.001	216			
**NPQ**	Species	2.49	0.83	3	0.12	**9.65**	**< 0.001**
	Light	148.47	29.69	5	0.89	**345.26**	**< 0.001**
	Species × Light	7.60	0.51	15	0.29	**5.89**	**< 0.001**
	Error	18.57	0.09	216			
**1-qP**	Species	0.61	0.20	3	0.48	**67.37**	**< 0.001**
	Light	2.79	0.56	5	0.81	**184.22**	**< 0.001**
	Species × Light	0.44	0.03	15	0.39	**9.58**	**< 0.001**
	Error	0.66	0.003	216			

Table S8 Ratio parameters O/P, P/S, P/M, S/M, M/T (mean ± SE, n = 10) for studied lichen species calculated from PSMT curves shown in Figure 5.

	**O/P**	**P/S**	**P/M**	**S/M**	**M/T**
**Light intensity 8 µmol photons m^{-2} s^{-1}**					
Cetrelia cetrarioides	0.741 ± 0.005	0.996 ± 0.007	0.863 ± 0.015	0.867 ± 0.013	1.560 ± 0.041
Flavoparmelia caperata	0.786 ± 0.006	1.019 ± 0.010	0.870 ± 0.030	0.853 ± 0.026	1.369 ± 0.056
Hypogymnia physodes	0.783 ± 0.006	1.028 ± 0.009	0.911 ± 0.019	0.886 ± 0.012	1.325 ± 0.025
Parmelia sulcata	0.789 ± 0.005	1.064 ± 0.007	0.927 ± 0.022	0.870 ± 0.017	1.345 ± 0.030
**Light intensity 500 µmol photons m^{-2} s^{-1}**					
Cetrelia cetrarioides	0.317 ± 0.004	1.159 ± 0.010	1.192 ± 0.011	1.029 ± 0.005	2.524 ± 0.121
Flavoparmelia caperata	0.336 ±0.010	1.690 ± 0.096	1.925 ± 0.101	1.139 ±0.017	1.777 ±0.088
Hypogymnia physodes	0.325 ± 0.007	1.272 ±0.035	1.336 ± 0.048	1.050 ± 0.011	2.395 ± 0.078
Parmelia sulcata	0.360 ± 0.005	1.095 ± 0.007	1.077 ± 0.008	0.983 ± 0.007	4.055 ± 0.124

References

Cieśliński S (2003) Atlas rozmieszczenia porostów (Lichenes) w Polsce Północno-Wschodniej. Phytocoenosis (NS). Suppl Cartograph Geobot 15:1–426

Genty B, Briantais JM, Baker NR (1989) The relationship between quantum yield of photosynthetic electron transport and quenching of chlorophyll fluorescence. Biochim Biophys Acta - Gen Subj 990:87–92

Horton P, Ruban AV (1992) Regulation of photosystem-II. Photosynth Res 34:375–385

Horton P, Ruban AV, Walters RG (1996) Regulation of light harvesting in green plants. Annu Rev Plant Physiol 47:655–684

Kalaji HM, Jajoo A, Oukarroum A, Brestic M, Zivcak M, Samborska IA, Cetner MD, Łukasik I, Goltsev V, Ladle RJ (2016) Chlorophyll a fluorescence as a tool to monitor physiological status of plants under abiotic stress conditions. Acta Physiol Plant 38:102

Lichtenthaler HK, Miehe JA (1997) Fluorescence imaging as a diagnostic tool for plant stress. Trends Plant Sci 2:316–320

Motiejūnaitė J, Czyżewska K, Cieśliński S (2004) Lichens – indicators of old-growth forests in biocentres of Lithuania and North-East Poland. Bot Lith 10:59–74

Nimis PL (2022) ITALIC - The Information System on Italian Lichens. Version 7.0. University of Trieste, Department of Biology, (https://dryades.units.it/italic), accessed on 2022, 09, 07

Paoli L, Pirintsos SA, Kotzabasis K, Pisani T, Navakoudis E, Loppi S (2010) Effects of ammonia from livestock farming on lichen photosynthesis. Environ Pollut 158:2258–2265

Rola K, Latkowska E, Myśliwa-Kurdziel B, Osyczka P (2019) Heavy-metal tolerance of photobiont in pioneer lichens inhabiting heavily polluted sites. Sci Total Environ 679:260–269

Wirth V (2010) Okologische Zeigerwerte von Flechten – erweiterte und aktualisierte Fassung. Herzogia 23:229–248
